# Norwegian energy community dataset: An electricity-hydrogen system with renewables, battery storage & hydrogen demand

**DOI:** 10.1016/j.dib.2025.112042

**Published:** 2025-09-09

**Authors:** Pratik Mochi, Magnus Korpås

**Affiliations:** Department of Electric Energy, Norwegian University of Science & Technology (NTNU), Trondheim 7034, Norway

**Keywords:** Green hydrogen, Electricity market, Energy market, Integrated energy system, Optimization model, Electrolyzer, Hydrogen market

## Abstract

This paper presents the Norwegian Energy Community Dataset, developed to support modelling and optimization of integrated electricity-hydrogen energy systems. The dataset represents a local energy community in Porsgrunn, Norway, consisting of 400 electricity end users, including residential, commercial, and industrial consumers and prosumers. It includes hourly smart meter data for electricity consumption and generation over a nine-month period (January to September 2024). The dataset features distributed energy resources such as rooftop solar PV (installed by 300 users), a 1.3 MW wind power plant and battery energy storage systems for 250 users. Electricity market data, including hourly buy and sell prices for 2024, are also included. On the hydrogen side, the dataset incorporates transport demand from (a) a real hydrogen-powered ferry operating in a northern Norwegian island and (b) 15 synthetic hydrogen buses modelled using Norwegian specifications and realistic operational patterns. Green hydrogen production is modelled using two electrolyzers (1.2 MW and 2.5 MW) along with associated hydrogen storage. The dataset would enable researchers to explore sector coupling, local energy market design, flexibility strategies and cost optimization of community scale integrated energy system.

Specifications Table

**Table:** 

Subject	Engineering & Materials science
Specific subject area	The data describes energy community consisting of energy end-users, renewables, battery, electrolyzer, hydrogen ferry & bus, electricity prices.
Type of data	Table (.xlsx format)
Data collection	Data from 25 PV users was scaled (× 0.8–1.2) to create 275 synthetic profiles. Wind data modelled for Nordex N60 turbine at Porsgrunn. Load data from 400 smart-metered users. 250 users assigned 8 real battery models. H₂ ferry demand based on prior inputs and fixed daily use. H₂ bus demand modelled via Julia, reflecting real ops, weather, and maintenance. Prices based on 2024 market/statistics. Grid limits set per user type (10–50 kW).
Data source location	City: Porsgrunn Country: Norway.
Data accessibility	Full dataset described in this publication Repository name: Zenodo Data identification number: https://doi.org/10.5281/zenodo.16778285 Direct URL to data: https://zenodo.org/records/16778285
Related research article	none

## 1 Value of the Data

• This dataset facilitates simulation of localized electricity markets integrated with hydrogen production and consumption. With real and synthetic data on consumption, generation, and hydrogen demand, researchers can investigate how localized market structures could support peer-to-peer energy trading and pricing strategies in communities. By designing internal market rules such as dynamic tariffs, real-time market clearing, or cooperative energy sharing researchers can explore ways to balance electricity and hydrogen flows. The interplay between electricity prices, hydrogen production via electrolyzers and demand from vehicles allows simulation of a truly integrated market. This work could influence regulatory models for community-based energy systems that include sector coupling
• The dataset includes distributed battery storage for 250 customers and two large electrolyzers. Future work can focus on co-optimizing their operation to achieve system-level goals such as cost minimization, peak shaving and emission reduction. Researchers can develop models that incorporate electricity market signals, user preferences, hydrogen demand profiles and technical constraints of batteries and electrolyzers. Joint optimization could consider when to charge/discharge batteries versus when to produce hydrogen. Additionally, strategies for demand-side flexibility such as storing energy during low-price hours and producing hydrogen during high renewable generation can be tested. This research is essential for demonstrating the benefits of hybrid energy systems.
• The dataset includes synthetic hydrogen demand profiles for a realistic hydrogen bus fleet operating on different routes. This enables researchers to simulate and analyze zero-emission public transit strategies under Norwegian conditions. Detailed route characteristics, refueling patterns and seasonal variation (including increased consumption in winter) are included. Studies can investigate optimal route planning, hydrogen refueling station placement, fleet scheduling, and total cost of ownership. Researchers can also simulate different fleet sizes, weather conditions, and load profiles to assess operational reliability and energy efficiency. This contributes to national and municipal planning for decarbonizing public transport
• Electrolyzers act as flexible loads that can respond to electricity market signals. This dataset supports modeling how electrolyzers can be dispatched in a price-responsive manner to minimize operational costs or provide grid services. Researchers can evaluate strategies that trigger hydrogen production during low-price or surplus renewable generation periods. This supports grid balancing and reduces curtailment of renewable energy. Additionally, the study can compare different electrolyzer control strategies such as rule-based versus optimization-based and their impacts on hydrogen availability and economics. This allows researchers to assess how time-sensitive hydrogen production may respond to fluctuating electricity prices and grid needs.
• This dataset allows simulation of isolated or weak-grid operation of an energy community. By introducing synthetic outage events or grid constraints, researchers can evaluate how the system can continue operation using local resources i.e. solar PV, batteries and hydrogen. Studies can test different resilience strategies, such as prioritizing critical loads, optimizing local generation, or coordinating distributed storage. Battery behavior under prolonged outages and the role of hydrogen as a long-term energy buffer can be analyzed. This supports the development of robust microgrid architectures for remote or vulnerable regions.
• Researchers can model the complete supply chain of green hydrogen using this dataset from electricity procurement to hydrogen production, storage and consumption. The presence of ferry and bus demand profiles enables detailed modeling of logistics and operational planning. Studies can include hydrogen storage management, transportation to refueling sites and matching of production with variable demand. Cost analysis can be performed under different electricity pricing regimes and electrolyzer capacities. This research supports the development of efficient, scalable hydrogen systems.

## 2 Background

Despite the increasing interest in integrated electricity-hydrogen systems, there is a lack of publicly available, high-resolution datasets that reflect realistic operating conditions at the local level. Such datasets are essential for researchers, planners and policymakers to develop, test, and validate models for system optimization, market design and policy analysis. Most existing datasets focus on national-scale data or lack detail on local consumption, generation, storage, and hydrogen usage. To address this gap, this paper presents the ``Norwegian Energy Community Dataset'' – a detailed dataset that captures the operation of a local energy system in the city of Porsgrunn, Norway, with both electricity and hydrogen components. This dataset models a local energy community that combines various energy consumption profiles with decentralized generation and storage. The system reflects the complexity of real-world energy environments where residential, commercial and industrial energy users operate alongside renewable resources and flexible technologies. With time-resolved data and realistic operating conditions, it provides a meaningful basis for analyzing demand behavior, local balancing and market interaction. In addition to electricity, the system also integrates hydrogen as a complementary energy carrier. Its role is primarily fulfilling hydrogen demand for transport applications, with local production and usage in connection to electricity availability.

## 3 Data Description

The dataset spans a 9-month period from January 1 to September 30, 2024 and includes hourly data across several categories. Each component is provided in .xlsx format, organized by timestep and a designated page name in excel sheet. The dataset structure is designed for ease of integration into simulation tools and optimization models. The data can be read in various programming languages e.g. JuMp, Python, MATLAB, R etc. The details of all pages in excel file are specified in Table 1. below.

**Table 1:** Dataset description.

Dataset Component	Description
Gen_pv	Hourly PV generation data for 300 (out of 400) users. Generated by scaling 25 base profiles using random multipliers (0.8–1.2) to simulate diverse capacities.
Gen_wind	Hourly wind generation for a 1.3 MW turbine using data from Renewables.ninja (Nordex N60 in Porsgrunn).
Load	Hourly electricity consumption from 400 users (real smart meter data including EV-driven peaks).
Buy_price	Hourly electricity purchase price for 2024
Sell_price	Derived electricity selling price based on Q3 2024 values from Statistics Norway (excluding taxes/fees).
Bat	Battery capacities and performance parameters for 250 users using 8 known battery models
H2_ferry_demand	Synthetic hourly hydrogen demand for 6 ferries in Norway.
H2_bus_demand	Synthetic hourly hydrogen demand based on daily bus operations (15 buses on weekdays, fewer on weekends).
Elz_storage	Specifications for two electrolyzers (1.2 MW and 2.5 MW) and associated hydrogen storage.
Grid_limits	Maximum power sell to Grid & Maximum power buy from Grid

## 4 Experimental Design, Materials and Methods

### 4.1 PV generation (Gen_pv)

The base data for 25 PV prosumers [1] was used to simulate a wider adoption scenario. To reflect varied system sizes and orientation, synthetic profiles for an additional 275 users were created by scaling the original 25 profiles using a random multiplier between 0.8 and 1.2. The direct irradiance data was not used in generating synthetic PV profiles & hence, variability can be observed by random multiplier. This approach helps represent a future-oriented community with widespread rooftop solar adoption.

### 4.2 Wind generation (Gen_wind)

Hourly wind generation data was obtained from [2] using a modeled Nordex N60 1.3 MW turbine. The selected site (Porsgrunn) and turbine parameters ensure that the generation profile reflects local weather conditions.

### 4.3 Load data (Load)

Smart meter data from 400 real users was used without modification [1]. The data includes a mixture of residential, commercial, and industrial demand. Visual inspection suggests the presence of EV charging in several household profiles, contributing to higher evening peaks.

### 4.4 Battery data (Bat)

To add flexibility to the energy community, battery systems were synthetically assigned to 250 users. Eight commercially available battery models [3] were selected and distributed randomly among users. Each model includes specifications such as capacity, charge/discharge rate and efficiency. The battery model details can be found in [3].

### 4.5 Hydrogen ferry demand (H2_ferry_demand)

The demand data for six hydrogen ferries was generated based on earlier inputs [4], assuming fixed routes and consistent daily usage patterns typical of Norwegian ferry operations. Each of the six ferries is assumed to operate a fixed route with daily hydrogen consumption set at 12 kg/day per ferry, reflecting typical mid-size ferry energy usage under average loading conditions. The dataset assumes consistent daily operation without seasonal adjustment, as route characteristics (e.g., distance, schedule) remain fixed year-round. Unlike buses, no weather-related correction factors were applied.

### 4.6 Hydrogen bus demand (H2_bus_demand)

Synthetic hydrogen bus demand was created based on realistic bus specifications, operational conditions & schedules [5,6]. A logical program was developed using Julia Mathematical Programming with following considerations.

• 15 buses run on weekdays, 10 on Saturdays, and 7 on Sundays.
• Buses are assigned to 3 fixed routes (30, 36 & 42 km roundtrip distance).
• Hydrogen consumption rate: 7.0 kg H₂ per 100 km. This consumption considers average traffic and passenger loading.
• Daily refueling occurs in one of three time windows (early morning, late morning, or afternoon).
• Cold-weather adjustments (+7 % consumption in Jan–Mar) and occasional shutdowns add realism. The national constitution day of Norway (17 May) is considered as holiday and hence no departures are scheduled.
• One bus is selected and undergoes for a full day for maintenance every month.

### 4.7 Electricity prices (Buy_price, sell_price)

Buy prices are based on real 2024 hourly market data [1]. Sell prices are estimated from Norwegian electricity statistics (Q3 2024), adjusted to exclude taxes and grid fees [7].

### 4.8 Maximum power import & export (Grid_limits)

For the variety of end-users in the low-medium voltage system, the maximum contracted power exchange limits are specified as 10 kW, 15 kW, 25 kW for residential and commercial buildings. For industrial plant, the contracted power limit is kept 50 kW. These limits are assumed to keep the power import and export within specified limit. The type of end user are also mapped with their ID.

### 4.9 Electrolyzer & storage (Elz_storage)

Hydrogen is produced locally using two electrolyzers: a 1.2 MW system inspired by the HyBalance project [[8], [9], [10], [11]] in Denmark and a 2.5 MW system modeled after the Haeolus project [12] in Norway. These electrolyzers are connected to the local electricity system and paired with hydrogen storage facilities, enabling time-shifted production and demand balancing. The specifications include electrolyzer production capacity, power rating, lower heat value, power range, electrolyzer efficiency, maximum and minimum hydrogen storage, charging and discharging efficiency of hydrogen storage and initially stored hydrogen. Detailed dynamic operational constraints such as ramp rates, start-up/shutdown time, or part-load efficiency curves are not explicitly modeled in the dataset. Users may implement such constraints during downstream optimization or control strategy development based on specific electrolyzer models. The dataset focuses on enabling flexible integration and time-resolved operation within local energy systems.

Some challenges in developing the dataset include limited access to real hydrogen demand data and simplifying assumptions in bus and ferry operations. Additionally, uncertainty in weather-driven parameters such as solar irradiance and wind speed is not explicitly modeled. These limitations are documented to support transparent usage. Future work could enhance this dataset by integrating dynamic weather data, stochastic modeling of hydrogen usage, and real-time grid interaction scenarios. Researchers may also expand on control strategies for electrolyzers and storage systems, evaluate demand response mechanisms, and validate synthetic profiles using real-world deployments.

The dataset spans January to September 2024, covering winter, spring, and summer conditions. Extending it to a full calendar year was not feasible due to data availability and modeling scope. However, users may build on this dataset to forecast the final quarter (October–December) using statistical or weather-driven models. This could form the basis for additional research on seasonal forecasting or uncertainty modeling in energy communities.

## Limitations

• PV profiles for additional users were generated by scaling real prosumer data, introducing diversity in system capacity and performance without requiring site-specific irradiance inputs.
• Hydrogen bus and ferry demand was synthesized using realistic vehicle specifications, fixed route lengths, seasonal consumption adjustments, and typical Norwegian transit behavior.
• Battery systems were randomly assigned based on real-world models, representing a variety of capacities and efficiencies across users.

## Ethics Statement

The current work does not involve human subjects, animal experiments, or any data collected from social media platforms.

## CRediT authorship contribution statement

**Pratik Mochi:** Conceptualization, Methodology, Software, Validation, Investigation, Resources, Data curation, Writing – original draft, Writing – review & editing. **Magnus Korpås:** Supervision, Conceptualization, Writing – review & editing, Formal analysis, Validation.

## Data Availability

zenodoNorwegian Energy Community Dataset for Local Electricity-Hydrogen System Modeling and Optimization (Original data).
